# MEMS Gyroscopes Based on Acoustic Sagnac Effect ^†^

**DOI:** 10.3390/mi8010002

**Published:** 2016-12-24

**Authors:** Yuanyuan Yu, Hao Luo, Buyun Chen, Jin Tao, Zhihong Feng, Hao Zhang, Wenlan Guo, Daihua Zhang

**Affiliations:** 1 State Key Laboratory of Precision Measurement Technology and Instruments, School of Precision Instruments and Opto-Electronics Engineering, Tianjin University, Tianjin 300072, China; yuanyuanyu@tju.edu.cn (Y.Y.); buyunc@tju.edu.cn (B.C.); taojin@tju.edu.cn (J.T.); zhfeng@tju.edu.cn (Z.F.); haozhang@tju.edu.cn (H.Z.); guowenlan@tju.edu.cn (W.G.); 2 Intel Labs, San Francisco, CA 95054, USA; memsluo@gmail.com

**Keywords:** acoustic gyroscope, Sagnac effect, phase difference, sound waves, angular velocity, sensitivity

## Abstract

This paper reports on the design, fabrication and preliminary test results of a novel microelectromechanical systems (MEMS) device—the acoustic gyroscope. The unique operating mechanism is based on the “acoustic version” of the Sagnac effect in fiber-optic gyros. The device measures the phase difference between two sound waves traveling in opposite directions, and correlates the signal to the angular velocity of the hosting frame. As sound travels significantly slower than light and develops a larger phase change within the same path length, the acoustic gyro can potentially outperform fiber-optic gyros in sensitivity and form factor. It also promises superior stability compared to vibratory MEMS gyros as the design contains no moving parts and is largely insensitive to mechanical stress or temperature. We have carried out systematic simulations and experiments, and developed a series of processes and design rules to implement the device.

## 1. Introduction

The gyroscope (gyro) is an inertial sensor determining the speed of rotational motions [1]. It has a wide range of applications including the fields of consumer electronics, automotive, aerospace and navigation [2,3,4]. Recently, gyros have witnessed a new wave of market growth driven by increasing needs of Internet of Things (IoT) and wearable devices. Their functions have been extended from basic motion sensing and navigation to much wider areas including human-machine interaction, health monitoring, and fitness analysis [5,6,7]. Among them, microelectromechanical systems (MEMS) vibratory gyros and fiber optic gyros are the most prevalent platforms in portable systems.

MEMS vibratory gyros are particularly attractive because of their small size, low cost, low power consumption, and easy integration with complementary metal-oxide-semiconductor (CMOS) circuitry [8,9,10]. They are based on the energy transfer between two orthogonal vibration modes as a result of the Coriolis effect. However, typical MEMS gyros come with a number of intrinsic drawbacks. The vibratory structure makes the device highly susceptible to external shock and vibration [11,12]. The structural design is inevitably associated with parasitic capacitive coupling and quadrature errors, which collectively deteriorate the performance and limit wider adoption of the MEMS gyros.

Fiber-optic gyros, on the other hand, are based upon a different sensing mechanism (the Sagnac effect), and do not involve the above issues. The device correlates angular speeds with interference signals from two laser beams. It usually consists of thousands of fiber coils to maximize the optical path length in order to achieve low drift and a high scale factor [13,14]. However, this generally makes the gyro too big and expensive for most consumer applications [12].

To overcome the drawbacks of the two classes of gyros, we propose a new MEMS device—the acoustic gyro—in this work. It is based on the “acoustic version” of the Sagnac effect in the fiber-optic gyro. The device measures the phase difference between two sound waves traveling in opposite directions in a circular air duct, and correlates the phase difference with the rotational speed of the waveguide. Since sound travels much slower than light, the acoustic gyro can develop a much larger phase difference within the same path length compared to fiber-optic gyros, or, equivalently, it can produce comparable sensing signals within a much shorter path length and significantly reduce the device size. In addition, the fabrication of the acoustic gyro is fully compatible with CMOS processes and it is readily integrated with peripheral electronics. In contrast to conventional MEMS gyros, the acoustic gyro is intrinsically immune to mechanical stress and temperature changes. The device also has a very simple structure that substantially lowers the manufacturing complexity.

This report expands on our previous work published in [15]. In this paper, we will present both the theoretical evaluations and the very recent progress in process development and testing. We have systematically evaluated and optimized a series of design rules to achieve acceptable process stability, device yield and performance. According to our theoretical predictions and preliminary testing results, the device can be packed into a 5 mm × 5 mm footprint and can potentially achieve a high sensitivity.

## 2. Materials and Methods 

### 2.1. Working Principle of Novel Device

**Figure 1:**
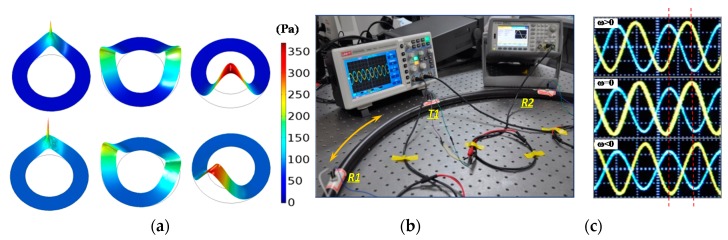
(**a**) Simulated sound wave propagating in a circular air duct when the air is stationary (above) and flowing (below). The setting of (below) is equivalent to the situation where the air keeps stationary while the frame (together with the sound transmitter and receiver(s)) rotates in the opposite direction. The out-of-plane displacement is the height expression of acoustic pressure (Pa); (**b**) Emulation test with a hula-hoop as the circular air duct. Diameter of the duct is about 80 cm. Three 40 kHz commercial ultrasonic ceramic transducers are installed inside the duct as acoustic transmitter and receivers. The transmitter and two receivers are marked as T1, R1 and R2, respectively; (**c**) Test results of (**b**). The phase difference between R1 and R2 changes when the ring rotates in clockwise and counterclockwise directions at ≈10°/s. The oscilloscope time base is 10 μs/div.

Figure 1 depicts the idea of the acoustic Sagnac effect in detail. A 3D finite element model (FEM) using COMSOL multiphysics software (v5.0, COMSOL Co., Ltd., Stockholm, Sweden) is set up to study the principle theoretically (Figure 1a). This model consists of a circular air duct, a sound transmitter, and a set of receivers. It couples the acoustic-piezoelectric interaction module and aeroacoustic module together. The former is used to simulate the sound transmitter and receivers with sound hard boundary condition, including sound generation and electro-acoustical conversion efficiency. The latter is adopted to emulate the pressure wave propagation in the air duct with no-slip boundary condition of air flow. The simulation results show the principle as below. When the transmitter at 12 o’clock position generates a pressure pulse, it induces two sound waves propagating in the clockwise and counterclockwise directions, respectively. When the transmitter, the duct and the air in it are stationary, the two wave fronts travel at the same speed and always meet at 6 and 12 o’clock positions (top in Figure 1a). On the other hand, if the duct and the transmitter move relative to the air inside or vice versa, the two sound waves propagate at different speeds (bottom in Figure 1a) and develop a phase difference with each other over time. Placing sound receivers along the air duct would allow us to quantify the phase difference and correlate the results with the rotating speed of the frame. The phase difference related with frame angular velocity can be detected by the subsequent digital phase detector. According to Reference [15], the phase shift Δφ and sensitivity $S_{\varphi }$ (defined as the phase shift per unit rotational velocity) of this device are

(1) $$\Delta \varphi =2\pi f\cdot \Delta t=\frac{8\pi^{2}R^{2}\Omega }{v^{2}}\cdot f$$

(2) $$S_{\varphi }=\Delta \varphi /\Omega =\frac{8\pi^{2}R^{2}}{v^{2}}\cdot f$$

where *R*, *f*, *v* and Ω are the radius of the air duct, acoustic frequency, acoustic velocity and the rotating speed of the frame, respectively. We can see from this formula the phase sensitivity is inversely proportional to the square of propagation velocity. The phase sensitivity between the acoustic and fiber-optic gyros can be calculated as

(3) $$\frac{\Delta \varphi_{\text{acoustic}}}{\Delta \varphi_{\text{optic}}}=\frac{f_{\text{acoustic}}}{f_{\text{optic}}}\cdot \frac{c_{\text{optic}}^{2}}{c_{\text{acoustic}}^{2}}=\frac{10\left[\text{MHZ}\right]}{193[\text{THz}]}\cdot \frac{{\left(3\times 10^{8}\left[m/s\right]\right)}^{2}}{{\left(343\left[m/s\right]\right)}^{2}}\approx 4\times 10^{4}$$

where *f*_optic_ is the light frequency using 193 THz [16,17,18], and *f*_acoustic_ is the sound frequency as 10 MHz which can propagated in air [19,20]. In this calculation we assumed the same ring radius *R* and angular velocity Ω for both devices. The estimation indicated that the acoustic gyro could theoretically achieve a phase sensitivity of 10^4^ higher than that of an optical device with the same dimension. In other words, the acoustic device could deliver comparable performance with significantly smaller size.

The Sagnac effect in optics derives from the assumption that the speed of light is independent of the motion of its source or medium. Apparently the same rule does not apply to ultrasound. In fact, the Acoustic Sagnac effect results from the inertia of air—the transmission medium keeps stationary when the circular duct rotates. Therefore, the two acoustic waves travel at the same speed in the inertial reference frame of the observer. This effect has been further verified by the emulation test in Figure 1c,d, in which a hula-hoop was used as the air duct. Three 12.6-mm-diameter 40 kHz commercial ultrasonic ceramic transducers (TCT40-12T/R-1.2) are installed inside the hula-hoop duct working as an acoustic transmitter and two receivers, respectively. The two ultrasound receivers located at different positions (labeled as R1 and R2) were able to detect different phase shifts when the duct was rotated at various speeds. The test result shows the time difference between the two acoustic waves is about 1.5 µs at an angular velocity of ~20°/s. It is in good agreement with the theoretical prediction.

### 2.2. Design and Structure

**Figure 2:**
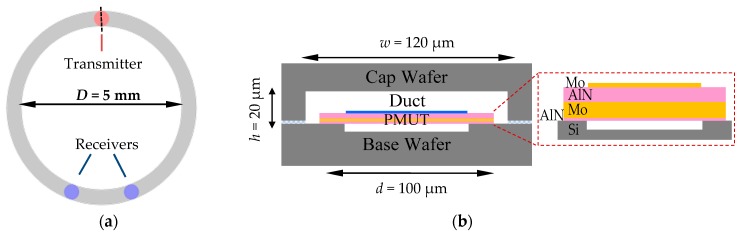
(**a**) Top-view schematic of the acoustic gyro (not in scale). In this specific case, three aluminum nitride piezoelectric micromachined ultrasonic transducers (AlN-PMUTs) are fabricated on the base wafer, one as a transmitter and two as receivers. The two receivers are placed symmetrically around the vertical axis. The circular trench on the cap wafer, once bonded to the base wafer, forms an enclosed air duct and bridges the three PMUTs; (**b**) Cross-sectional view across the center of the transmitter as marked by the dashed line in (**a**). The inset shows cross-section view of PMUT.

Figure 2 illustrates our scheme to build the gyro on a miniature MEMS chip. Figure 2a,b show the top and side views of the device with dimensions. The device is fabricated by bonding a cap wafer (containing a circular trench and through silicon vias (TSVs)) to a base wafer hosting a set of aluminum nitride piezoelectric micromachined ultrasonic transducers (AlN-PMUTs). The AlN-PMUTs are used as the acoustic transmitter as well as the receivers. The trench on the cap wafer defines the circular air duct to guide the acoustic waves. We selected PMUTs over capacitive micromachined ultrasonic transducers (CMUTs) due to the fact that the former does not need exceedingly high voltage bias (usually hundreds of volts for CMUT) or ultrafine microstructures to achieve sufficient transducer sensitivity, which effectively reduces circuit and fabrication complexity and cost [21,22,23].

Before assigning specific dimensions to each component, the operating frequency *f*_0_ needs to be determined first. It is related to several factors. According to Equation (1), higher frequency yields higher sensitivity of phase shift to rotational speed. However, it may also lead to larger acoustic attenuation, making the ultrasound waves hard to be detected at the receivers. In addition, one needs to consider the relationship between the wavelength (λ = *v*/*f*_0_) and the PMUTs’ diameter (*d*) as well. First, *d* needs to be smaller than λ to avoid near-field irregular pressure pattern [24]. Second, λ is proportional to the square of *d* as a result of flexural-mode resonance. Therefore, *d* needs to be greater than a lower limit to satisfy both requirements. This imposes an upper limit to *f*_0_. Taking into account all the requirements and limitations, we finally set the operating frequency *f*_0_ to be approximately 1.6 MHz. The diameter of PMUT should not be too small as compared to the wavelength for efficient transmission [25,26], or greater than the wavelength resulting in irregular near-field pressure pattern [24]. An ideal choice for the diameter of PMUTs in this work is 100 μm [25].

Given the target frequency (*f*_0_ = 1.6 MHz) and PMUT diameter, we are now able to determine the stack thickness of the PMUTs (*f*_0_ scales linearly with the film thickness and 1/*d*^2^ [25]) based on the given material. However, the layer thickness will impact other performance parameters such as electromechanical coupling coefficient, transmitting sensitivity (Pa/V) or receiving sensitivity (V/Pa). In this work, each PMUT on the base wafer consists of 4 functional layers suspended on an air cavity, which are the top electrode (TE, made of Mo), piezoelectric layer (PZ, made of AlN), bottom electrode (BE, made of Mo), and the seed layer (SL, made of AlN) from top to bottom. A big advantage of AlN is that its relatively low dielectric constant minimizes device capacitance, thereby producing a higher voltage between the top and bottom surfaces when operated as an ultrasonic receiver. The BE and SL layers serve as a passive layer to induce a vertical stress gradient across the whole stack, which forces the suspended membrane to deflect vertically when a transverse stress originates in the PZ layer (due to the piezoelectric coefficient e_31,f_). Different BE and SL thicknesses (with respect to the PZ layer) result in different transmitting and receiving sensitivities of the PMUTs. In our case, the net efficiency of the entire ultrasound generating, propagating, and receiving processes can be written as:

(4) $$S=\frac{v_{\text{output}}}{v_{\text{input}}}=\frac{P_{\text{tx}}}{v_{\text{input}}}\cdot \frac{v_{\text{output}}}{P_{\text{rx}}}\cdot \frac{P_{\text{rx}}}{P_{\text{tx}}}=G_{T}\cdot G_{R}\cdot G_{\text{ch}}$$

where *v*_input_ and *v*_output_ are the voltages applied to and detected at the transmitter and the receiver(s), respectively. *P*_tx_ and *P*_rx_ are the magnitude of the pressure wave right above the two PMUTs. The three parameters *G*_T_, *G*_R_ and *G*_ch_ characterize the transmitting sensitivity (in Pa/V), receiving sensitivity (in V/Pa) and the acoustic transmission attenuation, respectively. Previous studies [27,28,29,30] have observed non-monotonic dependence of *G*_T_ and *G*_R_ on the thickness of the passive layer. Qualitatively speaking, a thicker passive layer facilitates flexural bending of the entire membrane, but decreases the overall electromechanical coupling coefficient $k_{\text{eff}}^{2}$ at the same time [31,32,33].

**Figure 3:**
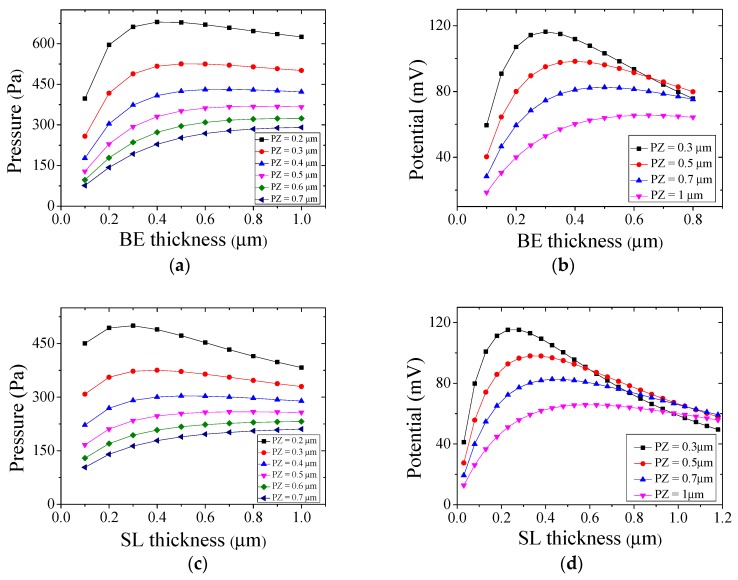
(**a**) Surface pressure under 1 V_pp_ driving voltage at the resonant frequency; (**b**) Electrical potential under 100 Pa as a function of piezoelectric layer/bottom electrode (PZ/BE) thickness ratio, with the seed layer (SL) thickness fixed at 50 nm; (**c**,**d**) The surface pressure and the electrical potential under various PZ/SL thickness ratios when the BE thickness is fixed at 100 nm.

We have setup a finite element model to quantitatively determine the optimum thickness. Figure 3a plots the surface pressure (in Pa) of the PMUT under a 1 V_pp_ driving voltage at resonant frequency as a function of PZ/BE thickness ratio. Peak position of each curve indicates the optimum thickness ratio for maximum *G*_T_. Figure 3b evaluates the receiving sensitivity for different PZ/BE stacks and plots the electrical potential developed under a constant pressure difference across the PMUT (100 Pa). The peaks correspond to maximum *G*_R_ values. Thickness of the SL is fixed at 50 nm in both figures. In Figure 3c,d, we repeat the same analysis with fixed BE thickness (100 nm) and varying PZ/SL ratios. According to the simulation results, multiply of the two coefficients (*G*_T_·*G*_R_) reaches its maximum at 500 (PZ)/500 (BE)/50 (SL) nm with the desired resonant frequency (1.6 MHz). This stack setting is used in our final design to optimize the net efficiency (*S*) of the transmitter-receiver pair.

It is worth noting that the top electrode (TE) of the PMUT has a smaller diameter compared to the rest layers in order to optimize the electromechanical coupling coefficient. We set the TE diameter as 70 μm, 70% of the PMUT diameter based upon the design rules discussed in previous reports [20,34,35].

We then use the following rules to set the geometric dimensions of the circular air duct. The channel width (*w*) should be shorter than the acoustic wavelength λ to avoid large sidelobes, and large enough to enclose individual PMUTs inside the channel (*w* > *d*). The perimeter of the circular duct (π*D*, *D* being the diameter) should be integer times of λ/2 to facilitate standing-wave formation. In addition, internal height (*h*) of the air duct needs to be sufficiently shorter than λ to minimize energy dissipation at boundaries. Based on these considerations, we set *w* = 130 μm, *D* = 5 mm, and *h* = 20 μm in our final design. 

**Figure 4:**
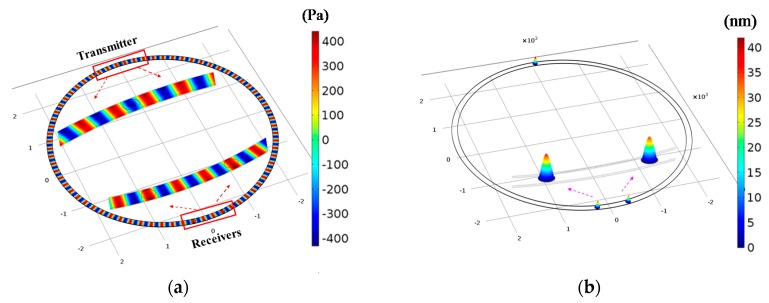
Finite element model (FEM) simulation results. (**a**) Acoustic pressure field distribution inside the air duct (*f*_0_ = 1.6 MHz). Color bar: Acoustic pressure (Pa); (**b**) Maximum mechanical displacement of the transmitter and receivers. Color bar: Displacement (nm).

Acoustic-piezoelectric frequency domain simulations are then carried out to verify the functionality of all components. In the model, we apply a continuous driving voltage of *V*_input_ = 10 V across the transmitter PMUT (at *f*_0_ = 1.6 MHz) and visualize the formation and detection of the acoustic waves. Figure 4a plots the distribution of the air pressure field, confirming the generation of a standing ultrasonic wave along the circular duct when the air duct is stationary. The inset zooms into the regions near the transmitter and the receivers to display finer features. Figure 4b examines the detection of the ultrasound when the air duct is stationary. It allows us to calculate the surface potential (*V*_output_) on the receiver PMUTs, which is around 65 mV in this specific setting. This corresponds to a net efficiency (*S*) of ~0.06 according to the definition in Equation (4). The simulation result demonstrates good detectability of the ultrasonic waves by the receivers, and proves good feasibility of our structural design.

**Figure 5:**
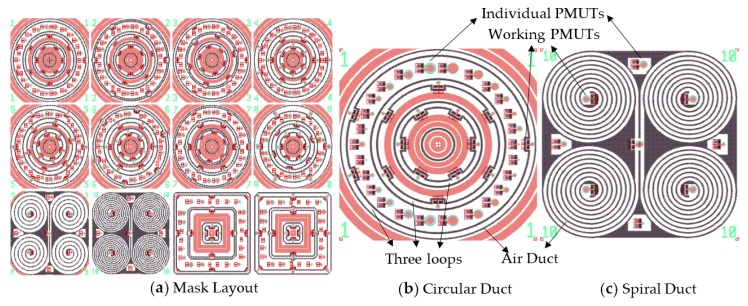
Mask layout with circular, spiral and square shaped air ducts. (**a**) Mask Layout; (**b**) Circular Duct and (**c**) Spiral Duct.

In addition to the above design, we have incorporated other design variations on the mask to maximize wafer usage. They include three kinds of ducts as circular, spiral and square. The circular ducts are designed within eight variants using different duct width, different PMUT diameter and quantities on duct. Two spiral ducts are presented by varying duct width and PMUTs diameter to improve device performance by maximizing path length. Each spiral shape includes four spiral structures which can eliminate cross sensitivity and the common–mode output errors by differential operation. The square ducts with different widths and PMUT diameters are used to evaluate possible dependence on the shape of the acoustic path. Figure 5 shows a section of the mask layout. The entire mask set contains 9 layers including all the PMUT, air duct, and TSV structures.

### 2.3. Fabrication

**Figure 6:**
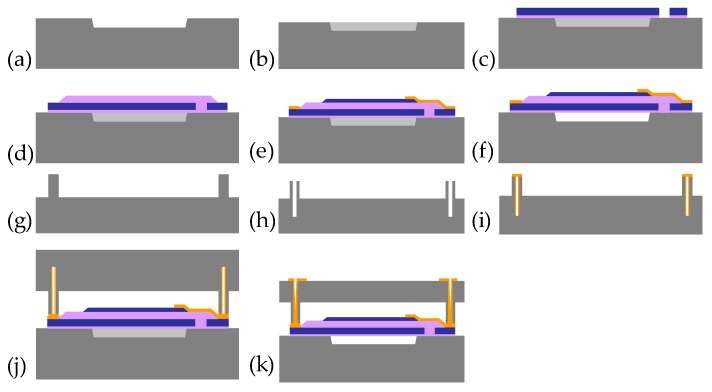
Base wafer flow: (**a**) Silicon etching; (**b**) SiO_2_ deposition and chemical mechanical planarization (CMP); (**c**) BE deposition and pattern; (**d**) AlN/TE deposition and pattern; (**e**) Au deposition and pattern; (**f**) sacrificial layer release; Cap wafer flow: (**g**) trench etch and pattern; (**h**) vias etch (**i**) 0.2 μm Cr/1 μm Au deposition and pattern; Bonding part: (**j**) Au-Au bonding; (**k**) CMP, electroplate, and Au deposition.

Figure 6 summarizes the entire process flow. The fabrication involves three major stages. The first stage deals with the fabrication of PMUTs on the base wafer (Figure 6a–f). Specifically, it starts with a sacrificial release pit formed by dry etching (Figure 6a) and CVD deposition of phosphosilicate glass (PSG) followed by chemical mechanical planarization (CMP) (Figure 6b). A 50 nm AlN seed layer and 500 nm Mo BE) are then deposited by sputtering at elevated temperature. The AlN seed layer provides a high-quality <110> crystalline foundation for the BE, PZ, and TE layers built on its top. It also electrically insulates the metal electrodes from the underlying Si substrate. The BE layer is then patterned by dry etch (Figure 6c). This is followed by deposition of 500 nm AlN and 150 nm Mo to form the PZ and TE layers, which are then patterned (through wet and dry etch to define the contours and expose metal pads and release holes (Figure 6d)). We use E-beam evaporation and lift-off to deposit Cr (0.2 μm)/Au (1 μm) pads in selected areas as the interface layer for wafer bonding (Figure 6e). In the final step, HF solution is used to remove the sacrificial layer and suspend the film (Figure 6f). 

Figure 6g–i depicts the process flow on the cap wafer. We first use dry etch to create a circular trench with side walls (Figure 6g), then make high-aspect-ratio holes through deep RIE (reactive ion etch) at the locations of TSVs (through silicon vias) (Figure 6h). The edges and inner walls of these holes are then covered with Cr (0.2 μm)/Au (1 μm) that works as the wafer bonding adhesive and the electrical interconnects between the base and the cap wafer (Figure 6i).

In the third stage, we flip over the cap wafer and bond it against the base wafer (Figure 6j). The bonding creates an enclosed air duct bridging all PMUTs on the same ring. Au-Au adhesion provides good bonding strength and establishes electrical connects between the two wafers at the same time. It is important to note that the bonding chamber is filled with N_2_ and maintained at an inner pressure of 1 atm throughout the process. The cap wafer also protects the PMUTs from damages by back-end processes including wafer dicing and plastic molding (when needed). The whole wafer is then thinned to 500 μm by mechanical grinding to expose the pre-defined TSVs. The vias are then filled with Cu by electroplating. This is followed by deposition and patterning of metal pads (Ti/Au) on the top surface to complete the entire flow (Figure 6k).

**Figure 7:**
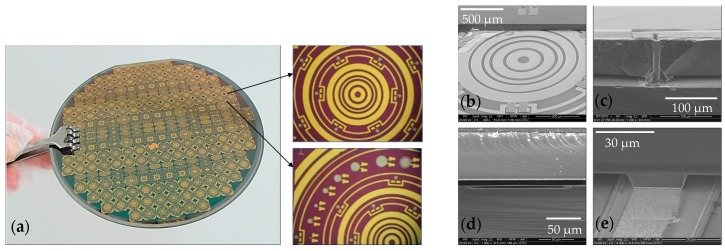
(**a**) Microscope images of whole wafer, circular device structure and individual PMUTs; (**b**) scanning electron microscope (SEM) image of a device with partially peeled-off cap; (**c**) close-up view of a TSV; (**d**) cross-sectional view of a PMUT; and (**e**) close-up view of a bonding interface.

The flow has proven to be a highly stable process and yields good uniformity and consistency. Figure 7a shows the microscope images of whole base wafer, circular device structure and individual PMUTs on it. Figure 7b–e are the scanning electron microscope (SEM) images taken from perspective angles to present close-up views of different structures.

## 3. Results

**Figure 8:**
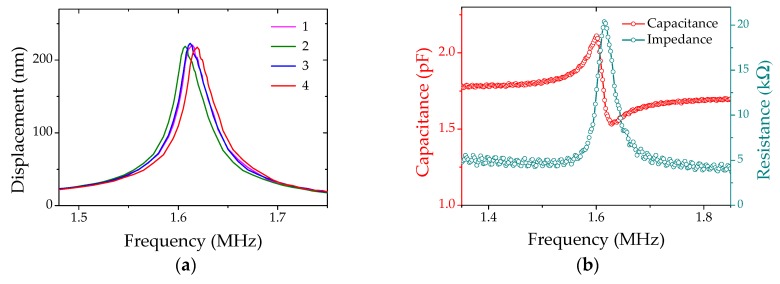
(**a**) Frequency dependence of surface displacements of four 100 µm PMUTs. The resonance frequencies are narrowly distributed with a very small variation of ~0.7%; (**b**) Impedance measurement of a single 100 um PMUT with a very high coupling coefficient of $k_{t}^{2}=3.64\%$.

We first evaluate the transmitting performance of the PMUTs. The surface displacement as a function of the driving frequency is measured in the air by a Laser Dropper Vibrometer (LDV, OFV 512 and OFV 2700, Polytec, Inc., Waldbronn, Germany) as shown in Figure 8. The PMUTs are excited with a 1 V sinusoidal signal. The measured resonant frequency is 1.616 MHz, very close to the designed value of 1.6 MHz. The maximum displacement at the resonant frequency is 221 nm. The measurements on four PMUTs (Figure 8a) at different locations indicate good frequency uniformity across the wafer. This ensures good frequency matching between the acoustic transmitter and receiver and maximizes the net efficiency *S*. The surface deflection (*d_f_*) is related to the local air pressure by

(5) $$P=(2\pi fd_{f})\rho_{0}c_{0}A_{e}$$

where ρ_0_ is the density of air, *c*_0_ is the sound velocity, and *A*_e_ = 1/3 is a correction factor accounting for the deviation from an ideal piston model [36,37]. The transmitting sensitivity *G*_T_ is then calculated to be ~330 Pa/V, slightly lower compared to the simulation result in Figure 3a, presumably due to geometrical mismatches and additional loss mechanisms through air damping, anchors and boundaries.

Impedance measurements of the PMUTs (100 µm in diameter) have been carried out using an impedance analyzer, the Agilent4294A (Agilent Technologies, Inc., Palo Alto, CA, USA, Figure 8b). The result shows good agreement on the resonant frequency with the LDV measurement. In addition, the impedance analysis provides both the resonance (*f*_r_) and anti-resonance *(f*_a_) frequencies, and allows us to calculate the electromechanical coupling coefficient $k_{\text{eff}}^{2}$ according to [23,38]

(6) $$\frac{k_{\text{eff}}^{2}}{1-k_{\text{eff}}^{2}}=\frac{f_{a}^{2}-f_{r}^{2}}{f_{r}^{2}}$$

Given *f*_r_ = 1.606 MHz and *f*_a_ = 1.636 MHz from the data in Figure 8b, $k_{\text{eff}}^{2}$ is calculated to be 3.64%. This value is significantly higher than typical numbers in previous reports on AlN-based PMUTs (0.056% in [23] and 0.387% in [39]). High material and process quality are likely acceptable for the improvement, as is using the mass production PVD tool for the film deposition. In addition, the device design also plays a role in this variance. The BE (Mo) layer serves as the passive layer with a high Young’s modulus. This increases the stiffness of the passive layer, thus raising the electromechanical coupling coefficient [40,41]. Moreover, the low parasitic capacitance in this PMUT contributes to the higher coupling factor as well.

**Figure 9:**
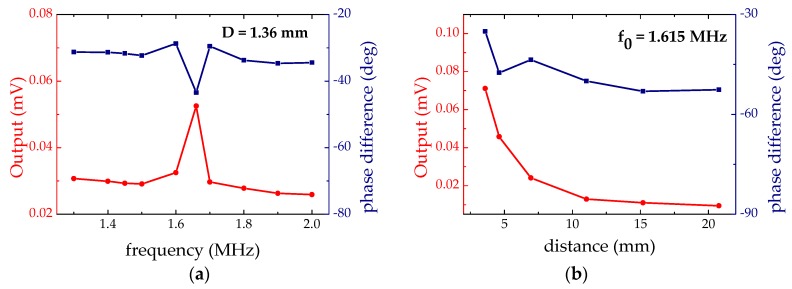
(**a**) Readout of the receiver at 1.36 mm from the transmitter; (**b**) Receiver signals taken at different distances from the source.

Next, we measure the receiving sensitivity by setting up two PMUTs facing each other. The two devices are wire-bonded to separate PCB boards, which are mounted on translational stages to continuously adjust the separation. The left PMUT is driven by a continuous sine wave (10 V_pp_) with a frequency swept from 1.3 to 2 MHz. The right is used as a receiver to pick up the ultrasound signal and feed it through a lock-in amplifier. Outputs of the amplifier, with both the amplitude and phase signals, are shown in Figure 9a. The distance between the two PMUTs is 1.36 mm. The maximum amplitude is about 52.5 µV at the resonant frequency of 1.617 MHz. The signal diminishes when we place thin sheets of dielectric materials (e.g., paper or glass) in front of the receiver, which confirms that the signal originates from the ultrasonic waves rather than the electromagnetic couplings. Furthermore, when we replace the dielectric sheets with a grounded metal mesh to block possible electromagnetic interference, the resonance signal persists. In Figure 9b, we record the receiver signals at different distances. The decay with increasing distance is in good agreement with acoustic attenuation in air.

## 4. Conclusions

In this paper, we present the design, fabrication and preliminary testing results of a new MEMS gyro. It measures angular velocity by comparing the phase difference between two acoustic waves. The device promises a similar performance to fiber-optic gyros with significant downsizing by four to five orders of magnitude. We have proven the concept by systematic FEM simulations, and successfully completed the fabrication and preliminary evaluation of the structural design. Further tests are ongoing to provide more comprehensive results and understanding of the design and sensing mechanism.
